# P19 Scarce supervision, strong pressures: systemic drivers of antibiotic prescribing by intern physicians in Bangladesh

**DOI:** 10.1093/jacamr/dlaf230.026

**Published:** 2025-12-04

**Authors:** Rakkan Afsara, Sumaia Shirin Humaira, Sanjida Khanom Chowa, Al Nuhin Hridoy, Nishat Hasan Emon

**Affiliations:** Department of Medicine, Sher-E-Bangla Medical College Hospital, Bangladesh; Department of Medical Oncology, Evercare Hospital Dhaka, Bangladesh; Department of Medicine, Faridpur Medical College Hospital, Bangladesh; Department of Medicine, Sher-E-Bangla Medical College, Bangladesh; Department of Medicine, Khulna Medical College, Bangladesh; MBBS 3rd year, Netrokona Medical College, Banglades

## Abstract

**Background:**

Antimicrobial resistance (AMR) is a global health crisis driven by the inappropriate use of antibiotics. Prescribing practices are often shaped during internship, and studies suggest that intern physicians typically follow attending physicians’ guidance when choosing antibiotics. However, evidence from a low- and middle-income country (LMIC) like Bangladesh remains limited. This study explored antibiotic prescribing practices and systemic drivers among intern physicians in Bangladesh.

**Methods:**

We conducted a cross-sectional survey between May and August 2025 in 13 public tertiary-care hospitals in Bangladesh. A structured, pre-tested questionnaire was administered in person and online to consenting intern physicians. The tool assessed timing of antibiotic initiation, empirical use, clinical triggers, routes of administration and systemic influences. Data were analysed using descriptive statistics.

**Results:**

Among 393 respondents, 330 (84%) reported prescribing antibiotics independently. A total of 71.2% initiated empirical therapy after clinical diagnosis but before laboratory confirmation, while only 9.7% routinely waited for culture results. Commonly cited reasons for empirical initiation were fear of treatment delays (54%), severe or worsening symptoms (48.1%), guidance from senior staff (28%) and hospital traditions (26.7%). The most frequent clinical symptoms prompting empirical use were fever (12.5%), wound infections (14.8%) and productive cough (8.4%). β-Lactam antibiotics were prescribed by 96.2% of intern physicians, while 27% reported prescribing fluoroquinolones irrespective of symptoms. Regarding administration routes, 80.4% reported prescribing IV, 64.9% orally and 45.3% both IV and orally. Systemic constraints strongly shaped prescribing choices. Pharmacy stock influenced antibiotic selection in 51% of cases. Time constraints due to heavy patient load were reported by 52.4% of interns, and hierarchical influence from senior physicians by 49.1%. Institutional support was limited: 257 interns (65.4%) reported no clear hospital antibiotic guidelines, and only 19.1% found existing guidelines easily accessible. Notably, 44.3% of interns indicated that accessible protocols would improve their practice.

**Conclusions:**

Intern physicians in Bangladeshi tertiary hospitals frequently prescribe antibiotics autonomously, often empirically and without microbiological confirmation. Their prescribing decisions are shaped less by evidence than by systemic and institutional pressures, including lack of guidelines, pharmacy shortages, time constraints and hierarchical expectations. The predominant use of broad-spectrum β-lactams further underscores risks for antimicrobial resistance escalation. Enhanced supervision, accessible guidelines, improved diagnostic capacity and pharmacy support are urgently needed to promote rational antibiotic use and strengthen stewardship.

